# Concurrent validity study of QuickDASH with respect to DASH in patients with traumatic upper extremity amputation

**DOI:** 10.1186/s12891-024-07183-w

**Published:** 2024-01-23

**Authors:** Joonas Pyörny, Ida Neergård Sletten, Jarkko Jokihaara

**Affiliations:** 1 Faculty of Medicine and Health Technology, Tampere University, Tampere, Finland; 2 Division of Orthopaedic Surgery, Oslo University Hospital, Oslo, Norway; 3 Center for Musculoskeletal Diseases, Tampere University Hospital, Tampere, Finland

**Keywords:** DASH, QuickDASH, Upper extremity, Amputation

## Abstract

**Background:**

The Disability of the Arm, Shoulder and Hand Outcome Measure (DASH) is a validated patient-reported outcome measure (PROM) for many upper extremity musculoskeletal disorders. In patients with severe traumatic conditions, limited evidence exists regarding the equivalence between DASH and its shortened version, QuickDASH, which is more feasible in clinical practice. The rationale of this study was to analyze the concurrent validity of QuickDASH with respect to DASH in patients with traumatic upper extremity amputation.

**Methods:**

This study is based on a consecutive cohort of traumatic upper extremity amputation patients treated with replantation or revision (completion) amputation at Tampere University Hospital between 2009 and 2019. We estimated the concurrent validity of QuickDASH with respect to DASH by correlation coefficients, mean score differences, Bland–Altman plots, and distribution density. Additionally, we assessed internal reliability with Cronbach’s alpha coefficients and item-total correlations.

**Results:**

We found a very strong linear correlation between DASH and QuickDASH scores (*r* = 0.97 [CI 95% 0.97–0.98], *p* < 0.001). The mean difference between DASH and QuickDASH was minor (MD = -1, SD 4 [CI95% from -1 to 0] *p* = 0.02). The mean sub-score for the activity domain was higher for QuickDASH than DASH (MD = -3 [CI95% from -4 to -3] *p* < 0.000) and lower for the symptom domain (MD = 7 [CI95% from 6 to 9] *p* < 0.000). The Bland and Altman plot showed good agreement between DASH and QuickDASH scores, but there was measurement error in QuickDASH with high scores (*r* = -0.20, [CI95% from -0.31 to -0.09], *p* = 0.001).

**Conclusion:**

QuickDASH demonstrates higher total scores than the full DASH and emphasizes rating of activity over symptoms. Still, on average the differences in total scores are likely less than the MCID of DASH, and consequently, this study shows that QuickDASH can be recommended instead of the full DASH when assessing a traumatic condition.

**Trial registration:**

Retrospectively registered.

## Introduction

The Disability of the Arm, Shoulder and Hand Outcome Measure (DASH) is a validated and well-established patient-reported outcome measure (PROM) for upper extremity physical disability and symptoms [1, 2]. DASH contains 30 items that evaluate disability and symptoms using a 5-step Likert scale (raw score from 1 to 5). The raw scores from each item’s score are transformed to a final score between 0 to 100, where a higher score indicates more disability and symptoms. To make the assessment more feasible, a shorter version (the QuickDASH) was created, which includes 11 items from the full DASH [3].

PROMs are generally considered the most important assessments after surgical interventions, particularly in musculoskeletal disorders [4–6]. Previous concurrent validation studies have shown a high equivalence between the original DASH and the QuickDASH scores in patients with non-traumatic upper extremity muscle disorders [7–10]. To our knowledge, there are only a few studies which have included some patients with traumatic disorders (upper extremity fractures) [11–14]. Based on previous reports, the DASH is considered to be an appropriate outcome for assessment after upper extremity amputations injuries [15–17]. The QuickDASH has been used for assessments in patients with upper extremity amputations [18–20], despite that there is no evidence of equivalence between the DASH and the QuickDASH in patients with severe traumatic injuries.

The primary aim of this study was to assess the concurrent validity of the QuickDASH with respect to the DASH in participants with traumatic upper extremity amputation. Secondary goals were to evaluate the cross-sectional validity and internal reliability between the DASH and the QuickDASH.

## Methods

### Study design and setting

This concurrent validity study includes a consecutive cohort of participants with traumatic upper extremity amputation who underwent replantation or revision amputation in Tampere University Hospital between 2009 and 2019. Data used in this present study are from the clinical studies of these patients.

### Participants and study size

The inclusion criterion was a traumatic upper extremity amputation that caused a fracture or exarticulation in the upper limb with loss of the circulation distal to the injury, excluding single-finger amputations. Participants who had not completed all the DASH items were excluded. There were no further exclusion criteria. The minimum follow-up time was 18 months (1.5 years). During the research period, a total of 372 participants met the inclusion. One participant (1/372) provided an incomplete answer for the DASH, and an additional 79 (79/372) did not respond. Resulting in a total of 292 (292/372, response rate 78%) participants included in the analysis for this study. The characteristics of the participants are presented in Table 1. Patients were sorted according to injury level: 1) distal to the carpus joint and 2) proximal to or through the carpus.

**Table 1:** Participants’ characteristics

	All patients (*N* = 292)
Age, mean (SD), years	56 (18)
Gender	
Male, n (%)	248 (85%)
Female, n (%)	44 (15%)
Level of injury	
Proximal to carpus, n (%)	31 (11%)
Distal to carpus, n (%)	261 (90%)
Completed answers	
DASH total, n (n/a)	286 (6)
DASH activity, n (n/a)	281 (11)
DASH symptoms, n (n/a)	289 (3)
QuickDASH total, n (n/a)	287 (5)
QuickDASH activity, n (n/a)	289 (3)
QuickDASH symptoms, n (n/a)	286 (6)

### Variables

Participants were asked to complete the validated Finnish translation of the DASH [21]. The full DASH has 30 items, while the QuickDASH includes 11 of them. All items are rated on a 5-step Likert scale [1, 3]. The optional work or leisure time domains of the DASH were not included in this study. After the participants had completed the full DASH, we calculated both the DASH and the QuickDASH scores, similarly as in previous validation studies [9, 11, 22]. To calculate a standardized score between 0 and 100, the full DASH requires a minimum of 27 completed items, while the QuickDASH requires a minimum of 10 completed items [23]. We also separately calculated raw scores for the activity and symptom items as separate domains (DASH questions 1–23 and 24–30, and QuickDASH questions 1–8 and 9–11, respectively). To calculate sub-scores for the activity and symptom domains, it is required that 90% of the items in each domain is completed: QuickDASH (7/8 for activity and 3/3 for symptoms) and DASH (22/24 for activity and for 5/6 symptoms) [10, 24]. The minimum clinically important difference (MCID) is estimated to 10 points (95% confidence interval [CI] from 7 to 14) for DASH [22] and to 14 points (95% confidence interval [CI] from 8 to 20) for QuickDASH [25].

For cross-sectional validation analyses, we used health-related quality of life by EQ-5D-5L index [26] (range from -0.62 to 1.0 with the Danish parameters, where -0.62 and 1.0 represent the worst and best health statuses possible), and EQ VAS (visual analog scale from 0; the worst imaginable health state to 100; the best imaginable health state); cold intolerance by the Cold Intolerance Symptom Severity (CISS) questionnaire (scale from 4 to 100 points, where a higher number indicates worse symptoms) [27] and global rating of upper extremity function on a numeric rating scale (NRS) from 0 to 10 (0 worst, 10 best). The outcomes used for cross-sectional validation were collected simultaneously from the participants with the DASH responses, chosen to cover various aspects of disability associated with severe post-traumatic conditions.

### Statistics

We used Pearson’s correlations, comparison of means (mean difference, MD) and the Bland–Altman analysis, a statistical method used to assess the agreement between two different instruments [28], to evaluate differences between the DASH and the QuickDASH scores. MD was calculated by subtracting the QuickDASH score from the DASH score. Cross-sectional validity was evaluated by the Pearson’s correlations between both DASH versions and the secondary outcomes. We used density plots to visualize the distribution of variables based on their density. Reliability (internal consistency and homogeneity) was analyzed with Cronbach’s alpha coefficients and item-total correlation (ITC), which describe the association of individual items with the mean of all other items, indicating the item validity in a questionnaire. An acceptable range for item-total correlation (ITC) in a multidimensional questionnaire is between 0.2 and 0.4 [29].

Continuous outcomes were presented as mean and standard deviation (SD). We used the paired t-test to compare mean DASH and QuickDASH scores and sub domain scores. The association between the two scores for each patient was measured using Pearson’s correlations and coefficients interpreted as follows: 0 to 0.19 as very weak, 0.20 to 0.39 as weak, 0.40 to 0.59 as moderate, 0.60 to 0.79 as strong, and 0.80 to 1 as very strong [30]. We set the significance level at α < 0.05.

## Results

**Fig. 1:**
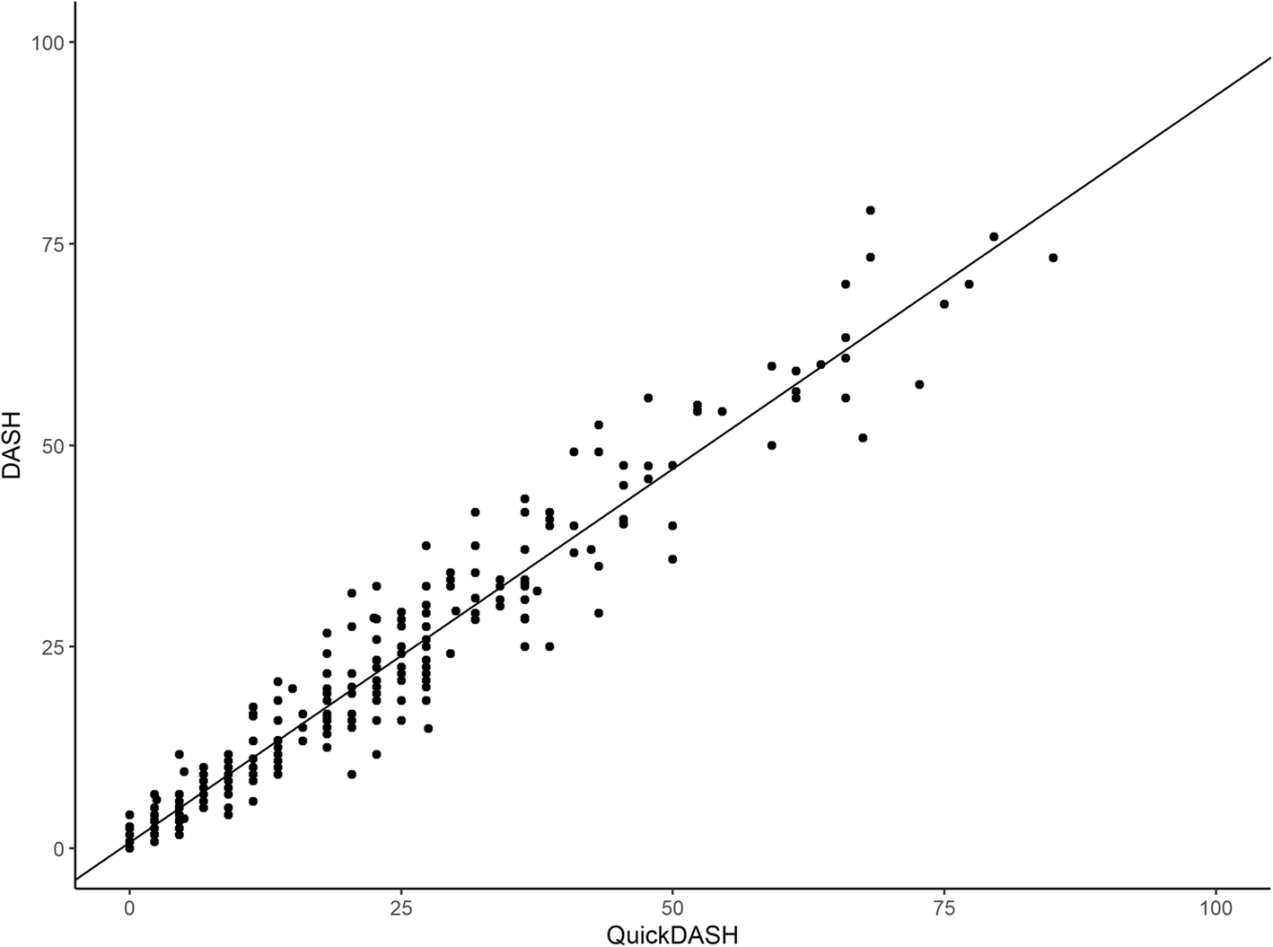
Scatter plot between the DASH and QuickDASH total scores

We found a strong linear correlation between DASH and QuickDASH scores; *r* = 0.97 (CI 95% 0.97–0.98, *p* < 0.001) (Fig. 1). The comparisons between mean DASH and QuickDASH scores show that the QuickDASH scores were slightly higher for the total group of participants and for participants with an injury level proximal to the carpus (Table 2). The mean sub-score for the activity domain was higher for QuickDASH than DASH and lower for the symptom domain (Table 3).

**Table 2:** Comparisons of mean DASH and QuickDASH scores according to injury level

Level of injury	DASH score mean (SD)	QuickDASH score mean (SD)	MD (95% CI)	p
All participants	19 (18)	20 (19)	-1 (-1 to 0)	0.02
Injury level distal to carpus	18 (17)	18 (18)	0 (-1 to 0)	0.10
Injury level proximal to carpus	33 (19)	36 (20)	-2 (-4 to 0)	0.03

DASH; All participants *N* = 286; distal to carpus *N* = 256; proximal to carpus *N* = 30

QuickDASH; All participants *N* = 287; distal to carpus *N* = 257; proximal to carpus *N* = 30

*Abbreviations*: *DASH* the Disabilities of the Arm, Shoulder, and Hand Outcome Measure (0–100, where 0 indicates no disability), *QuickDASH* the shortened version of the Disabilities of the Arm, Shoulder, and Hand Outcome Measure (0–100, where 0 indicates no disability), *SD* standard deviation, *MD* mean difference of DASH-QuickDASH, *CI* confidence interval

**Table 3:** Comparison of mean DASH and QuickDASH sub scores for the activity and symptoms domains according to injury level

	DASH activity sub score mean (SD)	QuickDASH activity sub score mean (SD)	MD (95% CI)	*p*	DASH symptoms sub score mean (SD)	QuickDASH symptoms sub score mean (SD)	MD (95% CI)	*p*
Level of injury								
Distal to carpus	16 (18)	19 (20)	-3 (-3 to -2)	< 0.000	24 (20)	16 (19)	7 (6 to 9)	< 0.000
Proximal to carpus	32 (20)	38 (21)	-5 (-7 to -3)	< 0.000	36 (22)	29 (27)	7 (1 to 12)	0.02
Total	18 (19)	21 (21)	-3 (-4 to -3)	< 0.000	25 (20)	18 (20)	7 (6 to 9)	< 0.000

Injury distal to carpus; DASH activity sub score *N* = 252; DASH symptoms sub score *N* = 258

Injury proximal to carpus; DASH activity sub score *N* = 29; DASH symptoms sub score *N* = 31

All participants; DASH activity sub score *N* = 281; DASH symptoms sub score *N* = 289

Injury distal to carpus; QuickDASH activity sub score *N* = 259; QuickDASH symptoms sub score *N* = 255

Injury proximal to carpus; QuickDASH activity sub score *N* = 30; QuickDASH symptoms sub score *N* = 31

All participants; QuickDASH activity sub score *N* = 289; QuickDASH symptoms sub score *N* = 286

*Abbreviations*: *DASH activity* score of 1–23 items from the DASH (0–100, where 0 indicates no disability), *QuickDASH activity* score of 1–8 items from the QuickDASH (0–100, where 0 indicates no disability), *DASH symptoms* score of 24–30 items from the DASH (0–100, where 0 indicates no symptoms), *QuickDASH symptoms* score of 9–11 items from the QuickDASH (0–100, where 0 indicates no symptoms), *SD* standard deviation, *MD* mean difference of DASH-QuickDASH, *CI* confidence interval

**Fig. 2:**
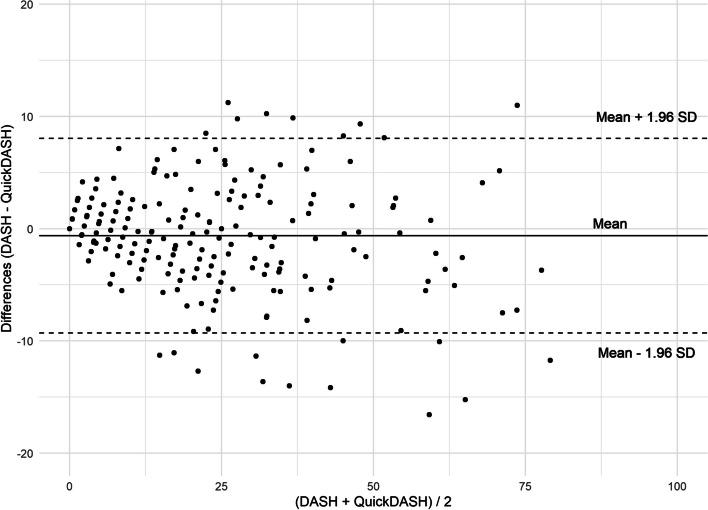
Bland and Altman plot (difference plot) for agreement between DASH and QuickDASH scores

**Fig. 3:**
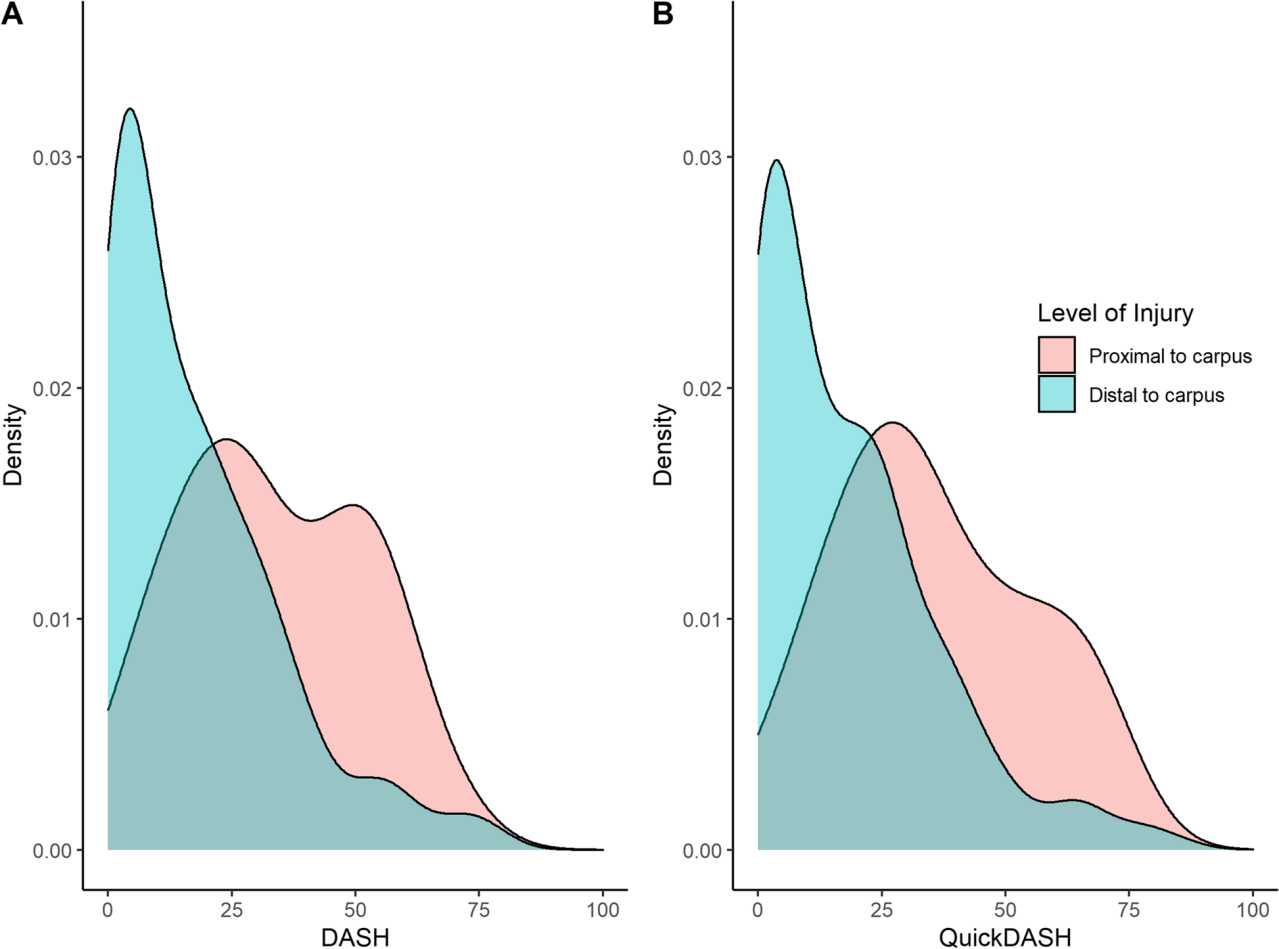
Density plots of (**A**) the DASH and (**B**) QuickDASH scores separated by injury level. Red color represents proximal to carpus injuries and blue represents distal to carpus injuries. A density plot visualizes the distribution of variables in terms of their density

The Bland and Altman plot (Fig. 2) showed good agreement between DASH and QuickDASH scores and most score differences (MD = -1, SD 4 [CI95% from -1 to 0] *p* = 0.02) were between the agreement limits (-0.6 ± 8.8 points). Absolute differences of 10 points or more were observed in 5% of patients (15/292), with score differences ranging from 11 to -17. The variance of differences was wider for higher scores, as indicated by correlation between the differences in DASH and QuickDASH scores and mean of DASH and QuickDASH (*r* = -0.20, [CI95% from -0.31 to -0.09], *p* = 0.001). Correlations between other PROM and the DASH and QuickDASH scores did not differ, indicating high cross-sectional validity (Table 4). The density distribution of the DASH and the QuickDASH scores indicated similar spreading of scores within the group of participants and a floor effect for both instruments in the group of participants with distal amputations (Fig. 3).

**Table 4:** Correlations between different outcome variables and the DASH or QuickDASH total scores

	DASH			QuickDASH		
	*r*	CI 95%	*p*	*0*	CI 95%	*p*
EQ-5D-5L index	-0.73	-0.78 to -0.66	< 0.000	-0.72	-0.77 to -0.66	< 0.000
EQ VAS	-0.58	-0.65 to -0.50	< 0.000	-0.55	-0.63 to -0.46	< 0.000
CISS	0.70	0.62 to 0.76	< 0.000	0.70	0.63 to 0.76	< 0.000
NRS of function	-0.54	-0.62 to -0.46	< 0.000	-0.56	-0.64 to -0.48	< 0.000

*Abbreviations*: *DASH* Disabilities of the Arm, Shoulder, and Hand Outcome Measure (0–100, where 0 indicates no disability), *EQ-5D index*, the EuroQol EQ-5D-5L index value (0–1, where 1 indicates the best situation), *EQ VAS* the EuroQol EQ-5D-5L health state with visual analogy scale value (0–100, where 100 indicates the best situation), CISS the Cold Intolerance Symptom Severity (0–100, where 0 indicates no symptoms), NRS (numerical rating scale) rating of function (0–10, where 10 indicates the best situation), *r* Pearsons correlation coefficient, *CI* confidence interval

Both DASH instruments had good internal reliability: Cronbach’s alpha value was 0.97 for the DASH and 0.92 for the QuickDASH. Item-total correlations (ITCs) for QuickDASH ranged from 0.55 to 0.79 and for DASH from 0.51 to 0.85. Of the five items with the highest ITCs in this study (DASH items 7,8,14,18, and 23), QuickDASH included all but one (item 8) (Table 5).

**Table 5:** Itemized DASH and QuickDASH scores, item-total correlations (ITCs), and correlations between DASH score and NRS (a numerical rating scale) rating of function

	DASH			QuickDASH
	ITC	Mean (SD)	NRS rating of function^a^	ITC
**1: Open a tight or new jar**	**0.75**	**2.1 (1.1)**	**-0.43**	**0.69**
2: Write	0.51	1.7 (1.2)	-0.25	
3: Turn a key	0.62	1.6 (1.0)	-0.24	
4: Prepare a meal	0.78	1.6 (0.9)	-0.39	
5: Push open a heavy door	0.74	1.4 (0.7)	-0.34	
6: Place an object on a shelf above your head	0.73	1.7 (1.0)	-0.39	
**7: Do heavy household chores (e.g., wash walls, wash floors)**	**0.85**	**1.8 (1.1)**	**-0.49**	**0.79**
8: Garden or do yard work	0.82	1.7 (1.0)	-0.49	
9: Make a bed	0.79	1.5 (0.8)	-0.40	
**10: Carry a shopping bag or briefcase**	**0.75**	**1.5 (0.8)**	**-0.38**	**0.69**
11: Carry a heavy object (over 10 lbs)	0.77	1.7 (1.0)	-0.42	
12: Change a lightbulb overhead	0.79	1.8 (1.1)	-0.36	
13: Wash or blow dry your hair	0.77	1.4 (0.9)	-0.34	
**14: Wash your back**	**0.80**	**1.8 (1.1)**	**-0.40**	**0.75**
15: Put on a pullover sweater	0.69	1.5 (0.8)	-0.34	
**16: Use a knife to cut food**	**0.73**	**1.9 (1.1)**	**-0.39**	**0.70**
17: Recreational activities which require little effort (e.g., cardplaying, knitting, etc.)	0.70	1.9 (1.1)	-0.35	
**18: Recreational activities in which you take some force or impact through your arm, shoulder or hand (e.g., golf, hammering, tennis, etc.)**	**0.81**	**2.1 (1.2)**	**-0.43**	**0.78**
19: Recreational activities in which you move your arm freely (e.g., playing frisbee, badminton, etc.)	0.79	2.0 (1.2)	-0.39	
20: Manage transportation needs (getting from one place to another)	0.67	1.3 (0.7)	-0.25	
21: Sexual activities	0.60	1.6 (1.1)	-0.33	
**22: During the past week, to what extent has your arm, shoulder or hand problem interfered with your normal social activities with family, friends, neighbours or groups?**	**0.62**	**1.4 (0.7)**	**-0.36**	**0.58**
**23: During the past week, were you limited in your work or other regular daily activities as a result of your arm, shoulder or hand problem?**	**0.80**	**2.1 (1.0)**	**-0.58**	**0.77**
**24: Arm, shoulder or hand pain**	**0.62**	**1.8 (0.9)**	**-0.45**	**0.67**
25: Arm, shoulder or hand pain when you performed any specific activity	0.62	2.0 (0.9)	-0.41	
**26: Tingling (pins and needles) in your arm, shoulder or hand**	**0.53**	**1.8 (1.0)**	**-0.36**	**0.57**
27: Weakness in your arm, shoulder or hand	0.67	2.1 (1.1)	-0.45	
28: Stiffness in your arm, shoulder or hand	0.64	2.2 (1.2)	-0.38	
**29: During the past week, how much difficulty have you had sleeping because of the pain in your arm, shoulder or hand?**	**0.53**	**1.5 (0.8)**	**-0.35**	**0.55**
30: I feel less capable, less confident or less useful because of my arm, shoulder or hand problem	0.58	2.6 (1.4)	-0.47	

The QuickDASH includes questions (1,7,10,14,16,18,22,23,24,26, and 29) (bold)

Answer options for DASH: items 1–21 (1 = No difficulty, 2 = Mild difficulty, 3 = Moderate Difficulty, 4 = Severe difficulty, and 5 = Unable); item 22 (1 = Not at all, 2 = Slightly, 3 = Moderately, 4 = Quite a bit, and 5 = Extremely); item 23 (1 = Not limited at all, 2 = Slightly limited, 3 = Moderately limited, 4 = Very limited, and 5 = Unable); items 24–28 (1 = None, 2 = Mild, 3 = Moderate, 4 = Severy, and 5 = Extreme); item 29 (1 = No difficulty, 2 = Mild difficulty, 3 = Moderate difficulty, 4 = Severe difficulty, and 5 = So much difficulty that I can’t sleep); item 30 (1 = Strongly disagree, 2 = Disagree, 3 = Neither agree nor disagree, 4 = Agree, and 5 = Strongly agree)

*Abbreviations*: *DASH* The Disabilities of the Arm, Shoulder, and Hand Outcome Measure, *QuickDASH* The Shortened version of the Disabilities of the Arm, Shoulder, and Hand Outcome Measure, *NRS* (numerical rating scale) rating of function, *SD* standard deviation, *ITC* Item-total correlation

^a^NRS rating of function: Pearson’s correlation coefficients between each DASH item and NRS (numerical rating scale) rating of function in injured upper extremity, *p*-values < 0.000

## Discussion

To test the concurrent validity between DASH and QuickDASH in traumatic musculoskeletal disorders, we evaluated DASH and QuickDASH scores after traumatic upper extremity amputation. Our study shows very strong correlations between the QuickDASH and DASH scores. Mean QuickDASH scores were higher than DASH scores, in particular in participants with amputations proximal to the carpus, but this difference was likely too small to be clinically meaningful. In addition, the mean sub-score for the functional disability domain was higher, and the mean sub-score for the symptoms domain was lower than for the full DASH, which means that QuickDASH overestimates functional disability and underestimates symptoms compared to DASH. QuickDASH showed good cross-sectional validity with other outcomes, similar to the full DASH. Our results support using the more feasible QuickDASH instead of DASH in patients with a severe traumatic condition, such as upper limb amputations.

We used the validated Finnish translation of the DASH [21], allowing us to generalize the results to all validated DASH translations. There is a potential source of bias related to our extraction of the QuickDASH items from the full DASH because we don’t know if the participants would have answered differently if they had completed solely the 11 items in the QuickDASH. We have not been able to address this bias, and we regard this as the major limitation of our study. We do, however, not regard this potential bias as disqualifying for our findings, but our results must be interpreted in relation to this aspect. The extraction approach, however, has been used in similar QuickDASH concurrent validation studies [7, 9, 11] and the wording of QuickDASH questions is exactly the same as in the full DASH. Another limitation is that our study was conducted at a single center and cultural factors, such as how participants emphasize functional disability over symptoms, may influence responses and limit the generalizability of the findings worldwide. Still, we had a relatively large sample size which decreased the uncertainty of results. In addition, our cohort included participants with a wide range of injury severity, from single thumb amputation to amputation proximal to the elbow, with a correspondingly wide range in DASH and QuickDASH scores.

The correlation between the DASH and the QuickDASH total scores was very strong in our study. It was our hypothesis, because QuickDASH questions are a carefully selected subset of the original DASH [9], and our study results on traumatic injury participants are in agreement with previous validation studies on other conditions [7–14]. We observed an overall slightly higher mean QuickDASH score than DASH score and previous studies have suggested similar findings on the mean score difference (from 1 to 5 points) [7–14] with upper extremity disorders. However, the mean difference in scores between DASH and QuickDASH was smaller than MCID, but nevertheless, 5% of patients had an absolute score difference equal to or higher than the MCID of DASH. The Bland and Altman analysis indicated generally good agreement between DASH and QuickDASH scores but showed that greater scores were associated with a wider variance of differences. This finding indicates greater uncertainty with QuickDASH in patients with more severe disabilities and symptoms.

The DASH is regarded as a suitable measure for evaluating outcomes following upper extremity amputation injuries [15–17]. Our study showed a floor effect with distal amputations, but it was less evident in proximal amputations, in which DASH scores distribution was closer to normal distribution shape. The floor effect of DASH with distal amputations may limit the sensitivity of the DASH to detect differences in patients who have only minor disability. Still, the moderate to strong correlations between the Quick-DASH or the DASH and secondary outcomes (EQ-5D-5L index, EQ-5D VAS, CISS, and NRS rating of function) indicate that both DASH instruments assess meaningful outcomes for patients after a traumatic upper extremity amputation.

Good Cronbach’s alpha values for both the Quick-DASH and the DASH indicate high internal consistency and this finding aligns with the previous studies [7, 10, 13, 14]. The QuickDASH showed consistently lower ITCs, which is in line with a previous report [7, 10]. However, the QuickDASH included four of the five items with the highest ITC in full DASH. This finding supports the developers’ statement that QuickDASH comprises the most important questions of the DASH for assessing upper extremity disability also after a severe traumatic injury.

This study demonstrates the usefulness of QuickDASH in patients with severe traumatic disorders. The instrument is less burdensome for both patients and assessors while validity is maintained. It is important to notice the measurement error related to higher scores, and different proportions of activity and symptoms assessment when compared with full DASH. However, on average the differences in total scores are likely less than the MCID of DASH or QuickDASH, and consequently, this study supports the recommendation to use QuickDASH instead of the full DASH when assessing traumatic conditions.

## Data Availability

The datasets used and/or analysed during the current study are available from the corresponding author on reasonable request.

## Abbreviations

DASH: The Disability of the Arm, Shoulder and Hand Outcome Measure
QuickDASH: The Shortened Disability of the Arm, Shoulder and Hand Outcome Measure
EQ-5D-5L index: The EuroQol EQ-5D-5L index value
EQ VAS: The EuroQol EQ-5D-5L health state with visual analogy scale
CISS: The Cold Intolerance Symptom Severity
NRS: Numeric pain rating scale
MCID: Minimal clinically important difference
ITCs: Item-total correlations
MD: Mean difference
CI: Confidence interval
r: Pearsons correlation coefficient
